# Supplementary material for: Genomic Correlates of APOBEC‐Associated Mutagenesis in Esophageal Squamous Cell Carcinoma
Source: Mol Genet Genomic Med. 2026 Nov 9;14(11):e70345. doi: 10.1002/mgg3.70345 (PMC13746264; doi:10.1002/mgg3.70345)
Supplement: Supplementary file 1 [file MGG3-14-e70345-s004.docx]

# Supplementary Figures

**Supplementary Figure S1. PCA sensitivity analysis.** Association between APOBEC proportion and PC1 before (A) and after (B) exclusion of SBS2 and SBS13. The association was lost after excluding the APOBEC-defining signatures.

## Supplementary Figure S2

Principal component analysis of mutational signatures according to country of origin. PCA plot demonstrating substantial overlap among tumors from different countries and the absence of distinct geographic clustering.
